# Penile emergencies: a review of the main conditions

**DOI:** 10.1590/0100-3984.2017.0072

**Published:** 2019

**Authors:** George Caldas Dantas, Francisco Cavalcante Jr., Fernando Ide Yamauchi, Marcelo C. J. Racy, Antônio Rahal Jr., Ronaldo Hueb Baroni

**Affiliations:** 1 Department of Radiology and Diagnostic Imaging, Hospital Israelita Albert Einstein, São Paulo, SP, Brazil.

**Keywords:** Penile diseases/pathology, Penile diseases/etiology, Emergencies, Emergency medicine, Urology, Radiology, Doenças do pênis/patologia, Doenças do pênis/etiologia, Emergências, Medicina de emergência, Urologia, Radiologia

## Abstract

Acute penile conditions, which typically have a traumatic, vascular, or infectious etiology, are rather uncommon and often require prompt medical evaluation. Penile emergencies can be treated conservatively or surgically, and their management often relies on the results of imaging examinations. Because of its high spatial resolution and wide availability, as well as the fact that it does not involve the use of ionizing radiation, ultrasound is the imaging modality of choice in the initial evaluation of penile emergencies. Inconclusive cases can be further evaluated with magnetic resonance imaging. The main purpose of this pictorial essay is to review the main penile emergencies, by presenting illustrative cases, focusing on radiologic findings, and discussing the roles played by the various imaging methods.

## Abstract (pt)

As condições agudas do pênis são raras, geralmente relacionadas a causas traumáticas, vasculares ou infecciosas, e muitas vezes requerem avaliação médica imediata. As emergências penianas podem ter tratamento conservador ou cirúrgico e muitas vezes dependem dos exames de imagem. A ultrassonografia é a modalidade de imagem de escolha na avaliação inicial das emergências penianas, em virtude da alta resolução espacial, ampla disponibilidade e ausência de radiação ionizante desse método. A ressonância magnética deve ser considerada em casos inconclusivos, oferecendo maior campo de visão e detalhes precisos sobre o local e extensão das lesões. O principal objetivo deste ensaio iconográfico é revisar as principais emergências do pênis por meio de casos ilustrativos, com foco nos achados radiológicos e no papel dos métodos de imagem.

## INTRODUCTION

Acute penile conditions are rather uncommon and can have a traumatic, vascular, or infectious etiology. The management of such conditions can be conservative or surgical and relies mainly on imaging findings.

As in evaluation of the scrotum, ultrasound is the first-line imaging modality, because it provides high spatial resolution, is widely available, and does not expose patients to ionizing radiation. In addition, color and spectral Doppler ultrasound can be used in order to evaluate vascularity. Magnetic resonance imaging (MRI) has come to play an increasingly greater role in patients with penile emergencies, particularly when the ultrasound findings are inconclusive. Because computed tomography lacks contrast resolution and involves the use of ionizing radiation, it should not be the method of choice in such cases.

The objective of this pictorial essay is to review the main penile emergencies. To that end, we present illustrative cases, focusing on the imaging findings and the role of each imaging method.

## PENILE EMERGENCIES

### Penile trauma

Penile trauma may be blunt or penetrating, and imaging is most often performed in the setting of blunt trauma. Penile fracture is related to a sudden pressure increase within the penile bodies, generally caused by an external force applied to the erect penis during vigorous sexual intercourse. Acute penile fracture manifests as an audible snap and sudden pain, followed by loss of erection, together with rapid swelling, generalized bruising, and deviation of the penis^(^^1^^,^^2^^)^.

**Figure 1:**
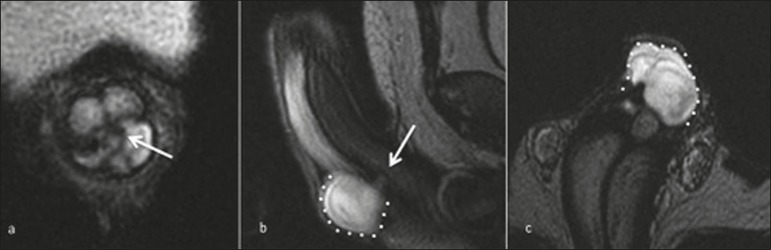
A 34-year-old man with penile pain after being kicked by a horse. T2-weighted MRI in axial (**a**), sagittal (**b**) and coronal (**c**) views showing a tear in the tunica albuginea (arrow) and an adjacent hematoma (dotted line).

**Figure 2:**
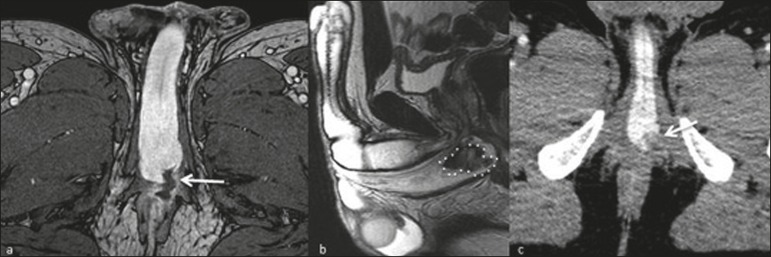
A 34-year-old man with a 10-day history of penile pain after a surfing accident. **a:** Contrast-enhanced axial T1-weighted MRI sequence showing a tear in the tunica albuginea extending to the corpus spongiosum and perineum. **b:** Sagittal T2-weighted MRI sequence showing a hematoma within the corpus spongiosum. **c:** An unenhanced computed tomography scan acquired 1 month later for the investigation of abdominal pain showing persistence of the hematoma within the corpus spongiosum.

Fracture usually occurs unilaterally in the distal two thirds of the penis. When present, hematoma is usually confined to the penis but can extend to the scrotum, perineum, and thighs. Imaging examinations are essential to evaluate the integrity of the tunica albuginea, a fibrous structure encapsulating each corpus cavernosum. Rupture of the tunica albuginea is characteristic of penile fracture and may occur in one or both corpora cavernosa (Figure 1) or, less commonly, in the corpus spongiosum (Figure 2), and surgical management is indicated in most cases. It is noteworthy that the tunica albuginea and Buck's fascia (the deep fascia of penis that covers all three bodies externally) are indistinguishable by imaging methods.

Ultrasound is the primary imaging method for the assessment of penile trauma, accurately demonstrating the normal anatomy and delineating the exact location and extent of the tear, which is seen as a thin echogenic line disrupting the tunica albuginea. A hematoma can be seen in the skin or deep in Buck's fascia and is helpful to identify the site of the tear, appearing as an anechoic area (hyperacute) or heterogeneous collection^(^^2^^,^^3^^)^.

**Figure 3:**
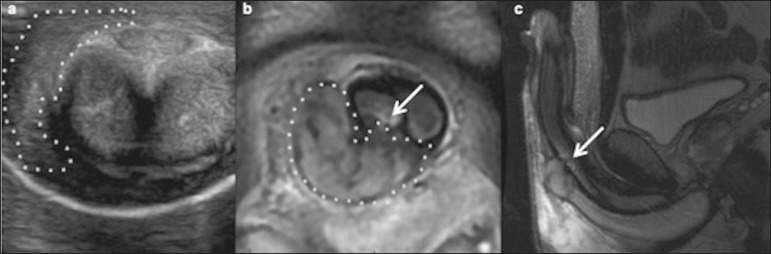
A 38-year-old man with penile pain and swelling after vigorous sexual intercourse. **a:** Axial ultrasound showing a hematoma (dotted line) adjacent to the right corpus cavernosum; no fracture was identified in the tunica albuginea. Coronal and sagittal T2-weighted MRI sequences (**b** and **c**, respectively) showing disruption of the tunica albuginea (arrows) and the adjacent hematoma (dotted line).

In cases of penile trauma, especially those in which there is a small tear, MRI can be performed if the ultrasound findings are inconclusive. A tear in the tunica albuginea is best evaluated on T2-weighted sequences dedicated to penile evaluation and appears as discontinuance of the hypointense signal of the tunica, that may be accompanied by intracavernosal or extratunical hematoma^(^^1^^)^, as depicted in Figure 3.

**Figure 4:**
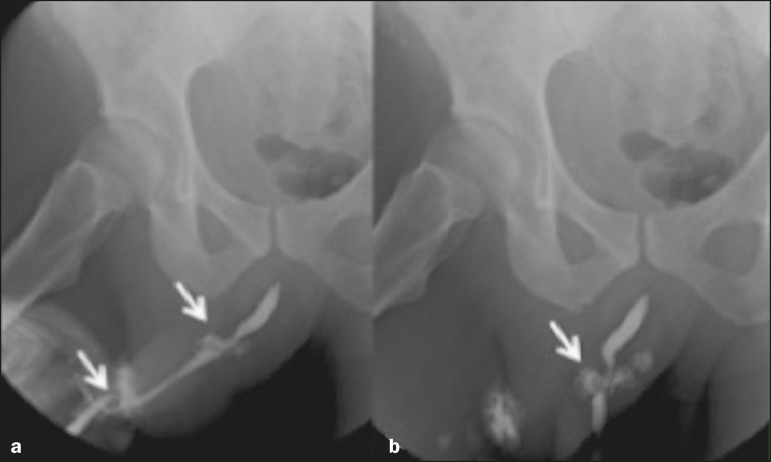
A 28-year-old man with a history of a tear in the tunica albuginea. **a,b:** Retrograde urethrography showing tears in the bulbar and penile urethra (arrows).

In cases of traumatic injury of the penis, the urethra can also be involved, particularly in its relatively fixed portion around the urogenital diaphragm. Although ultrasound evaluation is quite limited, the presence of hyperechoic foci in the corpora cavernosa, suggestive of air foci, may indicate urethral injury and should prompt further investigation^(^^4^^)^. In penile trauma with hematuria or urinary retention, retrograde urethrography is always indicated for the evaluation of a urethral tear (Figure 4).

Urgent surgical repair is usually necessary in cases of a tear in the tunica albuginea, and identifying the exact location of the fracture allows better surgical planning and localized surgical exploration. Conservative management can be an option in selected cases, particularly in patients who have small tears without hematoma or urethral injury^(^^2^^)^.

### Priapism

Priapism is a pathological and often painful prolonged penile erection, unassociated with or persisting beyond sexual stimulation. It is classified as one of two types^(^^5^^)^: low-flow (ischemic) or high-flow (arterial or non-ischemic).

Low-flow priapism is the most common type. It results from impaired penile venous drainage that promotes high cavernosal pressures and, if sustained, can lead to irreversible ischemic changes and permanent erectile dysfunction. Possible etiologies include prothrombotic states (such as sickle cell anemia), neoplastic disease, and medication use. Low-flow priapism is a urological emergency.

High-flow priapism involves dysregulation of penile blood flow and is usually secondary to an injury or surgical trauma^(^^5^^)^. It is not considered an emergency, because it is rarely associated with pain, ischemia, or permanent erectile dysfunction^(^^6^^)^.

**Figure 5:**
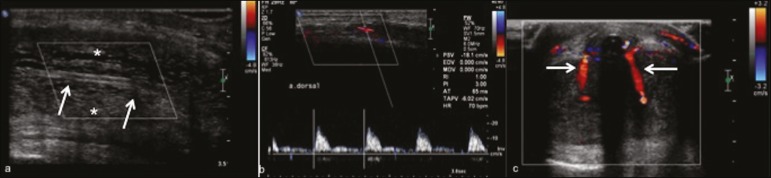
A 36-year-old man who had had a mildly painful erection for three days. **a:** Longitudinal Doppler ultrasound showing increased echogenicity of the corpora cavernosa (asterisks), consistent with tissue edema, and obliteration of the cavernosal arteries (arrows) with no flow on the color Doppler study. **b:** Duplex scan showing high flow resistance at the dorsal artery of the penis, consistent with low-flow (ischemic) priapism. **c:** Postoperative axial Doppler ultrasound showing a surgical shunt between the corpus cavernosum and the corpus spongiosum at the glans penis.

Low- and high-flow priapism differ in clinical and biochemical aspects. Low-flow priapism is painful and acute, resulting in rigidity, whereas high-flow priapism usually manifests as a painless prolonged erection after an incomplete trauma. On Doppler ultrasound, low-flow priapism may show high resistance to or even the absence of flow in the cavernosal arteries (Figure 5). In such cases, the goal of therapy is to reverse the ischemic insult quickly, and the first-line options are administration of phenylephrine and direct corporal aspiration. In high-flow priapism, Doppler may show high blood flow with a pattern of low resistance in the cavernosal arteries, a pseudoaneurysm, or an arteriocavernosal fistula^(^^5^^-^^7^^)^.

Although not routinely performed in the setting of priapism, penile MRI may indicate a worse prognosis. In the corpora cavernosa, signal loss in T2-weighted sequences, followed by a lack of enhancement in contrast-enhanced T1-weighted sequences, indicates nonperfusion or patchy perfusion suggestive of fibrosis and smooth muscle necrosis^(^^8^^)^. Permanent erectile dysfunction is a long-term sequelae due to progressive fibrosis of the corpora cavernosa, ultimately requiring implantation of a penile prosthesis.

For suspected nonischemic priapism, penile angiography can be diagnostic, revealing an arteriovenous fistula, and therapeutic, allowing super-selective embolization aimed at disrupting the aberrant vascular connection or occluding the cavernosal artery. Complications of embolization, such as penile gangrene, gluteal ischemia, and erectile dysfunction, may occur^(^^6^^)^.

Erectile dysfunction is a long-term sequelae for these patients due to progressive fibrosis of the corpora cavernosa. and ultimately requiring penile implantation many times in young, sexually active patients may ultimately implantation of a penile prosthesis.

### Penile Mondor's disease

Penile Mondor's disease, also known as penile superficial venous thrombosis, is a rare, self-limiting process, characterized by acute thrombosis in the superficial dorsal vein of the penis, that typically affects sexually active young men. Clinically, it presents as a palpable cord in the dorsal vein of the penis, with pain or local discomfort, especially during erection. Although the etiology is poorly understood, causes of the disease include prolonged sexual intercourse or abstinence, penile trauma, local or distant infections, pelvic surgery, and a hypercoagulable state^(^^9^^)^.

**Figure 6:**
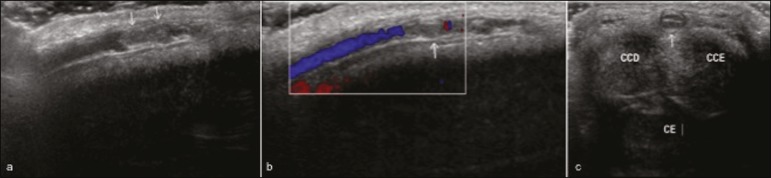
A 53-year-old man, with no relevant medical history, complaining of penile pain. Longitudinal B-mode ultrasound showing an echogenic thrombus within the superficial dorsal vein of the penis (arrows in **a**) with no flow on the color Doppler study (**b**). Axial B-mode ultrasound (**c**) showing thrombosis of the superficial dorsal vein of the penis. Note the increased caliber of the vein, which is filled with echogenic material (**c**). CCD, right corpus cavernosum; CCE, left corpus cavernosum; CE, corpus spongiosum.

Penile Mondor's disease may be diagnosed on the basis of the medical history and physical examination findings. Color Doppler establishes the diagnosis and can be used in order to monitor the disease. A lack of blood flow, the absence of normal compressibility, and the presence of an intraluminal thrombus indicate dorsal vein thrombosis (Figure 6). The use of MRI has also been reported, although only in complex cases and cases in which the initial findings are inconclusive^(^^9^^,^^10^^)^.

### Infection

**Figure 7:**
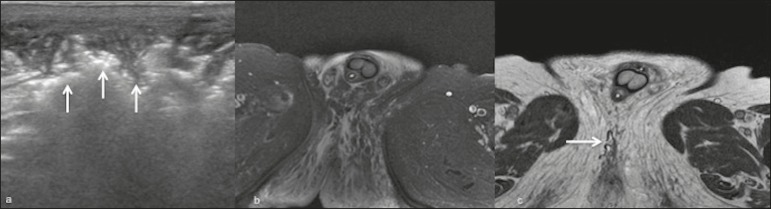
An 83-year-old man with pain and edema in the scrotum and perineal region. **a:** Ultrasound showing thickening and heterogeneity of the skin and subcutaneous tissue of the scrotum and perineal region, with hyperechoic foci and posterior acoustic shadowing (arrows), suggesting gas foci. **b:** Axial T2-weighted MRI sequence with fat suppression, showing extensive edema in the perineal region, with gas foci (arrows) that were more evident on an axial T1-weighted sequence (**c**).

Penile infections can be divided into superficial and deep infections. Superficial infections are the most common and are restricted to the glans and prepuce (balanitis and balanoposthitis, respectively). Such infections are often related to poor personal hygiene or are sexually transmitted; they are managed clinically, and imaging evaluation is unnecessary^(^^11^^)^. A deep penile infection, also known as Fournier's gangrene, constitutes a urological emergency, typically presenting as rapidly progressing, life-threatening necrotizing fasciitis of the perineal, perianal, and genital regions, including the penis. It is a rare condition with a wide spectrum of presentation. Although the diagnosis is often made clinically, radiologic imaging is useful for assessing the extent of the disease and facilitating the surgical planning. In this particular scenario, computed tomography is preferred over MRI. Imaging findings include subcutaneous and fascial thickening accompanied by fat stranding, fluid collection, or abscess. Subcutaneous emphysema secondary to gas-forming bacteria is the most specific sign. Ultrasound plays a limited role in these cases, although it can show soft-tissue thickening and identify hyperechoic foci with "dirty" shadowing suspicious for emphysema^(^^12^^)^, as shown in Figure 7.

## CONCLUSION

Although uncommon, penile emergencies require prompt, accurate diagnosis. Ultrasound is the primary imaging modality for the initial evaluation, and MRI may be useful in selected cases. Familiarity with the main imaging findings allows a correct diagnosis and facilitates further evaluation with other imaging methods when appropriate.
